# Health warning labels describing snus as less harmful than smoking: effects on perceptions of risk

**DOI:** 10.1186/s12954-020-00380-5

**Published:** 2020-06-05

**Authors:** Connie Villemo Nilsen, Torleif Halkjelsvik, Frode Svartdal

**Affiliations:** 1 Department of Psychology, Faculty of Health Sciences, UiT The Arctic University of Norway, 9037 Tromsø, Norway; 2 Department of Alcohol, Tobacco and Drugs, Norwegian Institute of Public Health, P.O. Box 222 Skoeyen, 0213 Oslo, Norway; 3 Centre for Evaluation of Public Health Measures, Norwegian Institute of Public Health, P.O. Box 222 Skoeyen, 0213 Oslo, Norway

**Keywords:** Snus, Smoking, Health warning labels, Comparative information, Perceived risk

## Abstract

**Background:**

Using snus (Swedish moist snuff) is less harmful than smoking, but health warning labels (HWLs) on snus products do not reflect this relation. There are few studies on the effects of comparative risk information in snus warning labels. The purpose of this experiment is to examine whether risk perceptions differ after exposure to non-comparative vs. comparative risk information in snus warning labels.

**Methods:**

A total of 254 Norwegians aged 19–69 were exposed to pictures of snus packages in one of four HWL conditions: non-comparative EU-based (“Snus is damaging to your health”), control (the text “Snus” only), general comparative risk (“Snus is less damaging to your health than smoking”), or percentage comparative risk (“Snus is 90% less damaging to your health than smoking”). Perceptions of risk from snus use and smoking were measured before (pre) and during (post) exposure to the HWL. Changes from pre to post in (1) perceptions of risk from snus use and (2) perceptions of risk differences from snus use versus smoking were tested in repeated measures ANOVAs with current snus and cigarette use as covariates.

**Results:**

Both the perceived risks from snus use and its perceived risk difference to smoking decreased more in the control and the percentage HWL conditions than in the EU-based HWL condition. When comparing the general comparative risk and the EU-based HWL, a similar difference was found for the perceived risk difference, but not for the separate measure of snus risk. Both the snus risk and risk difference perception decreased more for the percentage than for the general relative risk HWL.

**Conclusions:**

The non-comparative EU-based HWL claiming that “Snus is health damaging” maintains a high level of perceived risk from snus use, while no HWL and the suggested comparative HWLs adjust perceptions of risk in the direction of lower harm from snus use. An HWL describing snus as 90% less harmful than smoking was more effective than a general claim.

## Background

Smokeless tobacco has long been proposed as a low-risk alternative to cigarettes. Recently, the U.S. Food and Drug Administration (FDA) concluded that completely switching from smoking cigarettes to using certain snus products lowers health risks [1]. As an implication, a specific brand of moist snuff (hereafter: “snus,” the Swedish-language word for the category) may now be sold in the U.S. with a modified risk claim that compares snus use to smoking: “Using General Snus instead of cigarettes puts you at a lower risk of mouth cancer, heart disease, lung cancer, stroke, emphysema, and chronic bronchitis.”

The public tends to exaggerate risks from using snus compared to risks from smoking cigarettes. Wackowski et al. [2] found that 74.6% of the U.S. smokers in their sample perceived snus to be as harmful or more harmful than smoking cigarettes. Even in Norway, where the number of daily snus users exceeded the number of daily smokers in 2017, the perceptions among lay people does not appear to reflect the different risk profiles of snus and cigarette smoking. In a study using survey data from Norwegians aged 16–79 in the years 2003–2018, the harmfulness of daily snus use was rated only somewhat lower than daily smoking. These perceptions did not appear to have changed during the last 16 years [3]. As for specific diseases, another study found that the majority of Norwegian smokers estimated that the risk for cardiovascular disease, oral and stomach cancer was equal or higher for snus users [4].

One way of communicating the risk of tobacco to users and potential users is through Health Warning Labels (HWL) on the product itself. HWLs are short statements about health risks, or pictures illustrating severe consequences associated with using a product, that are applied on products to communicate. HWLs are a part of the tobacco control measures described in the World Health Organizations Framework Convention on Tobacco Control [5], which was developed to reduce the harms from tobacco. Cigarette packages are subject to more restrictive regulations compared to smokeless tobacco products (SLT), (e.g., to carry larger, graphic HWLs), which may reduce initiation and increase smoking cessation more than textual HWLs [6, 7].

Tobacco regulations for SLT products typically include textual warnings [7]. An HWL regarding risk of cancer, “This tobacco product severely damages your health and is addictive. Causes cancer” [8], was removed from snus products sold within the European Union (EU) in 2003 (snus sale was only allowed in Sweden at that time), and replaced with a more general warning: “This tobacco product can damage your health and is addictive” [9]. The HWL was modified slightly in 2016, when the modal verb “can” was removed [10]: “This tobacco product damages your health and is addictive.” This new statement was expected to strengthen the risk message, but the effect may have been minor [11].

Given the reduced health risks from SLT compared to cigarettes and their potential as a harm reduced alternative to smoking, it has been argued that product information should reflect SLTs risks in comparison to smoking [12]. Alternatives to cigarettes, such as snus, may be perceived as more favorable if the HWLs concern health risks relative to the risks of smoking, because cigarettes are a product that most people know is very harmful [13]. It is therefore interesting from a harm reduction perspective to understand how such comparative HWLs affect risk perception and behavior [13–15].

To our knowledge, only a few studies have exposed participants to comparative HWLs on SLT products or models of such products. Note that messages comparing risks are typically referred to as “health warning labels” because of the shared format with standard HWLs, although the information strictly speaking concerns comparative information. In an online experiment, Canadian smokers exposed to the HWL “Using ST is less harmful than smoking cigarettes” more often reported perceptions of risk that reflected the large difference in risks between SLT and smoking than those who were exposed to regular HWLs [16]. The youths who were exposed to the comparative HWL also had a higher likelihood of reporting future use of SLT, and a higher willingness to try SLT as a cessation aid. The comparative HWL was designed for experimental purposes and is not applied on SLT packages in Canada.

In a study sponsored by the snus manufacturer Swedish Match, participants were exposed to one of four current US HWLs, and two proposed comparative risk HWLs: “No tobacco product is safe, but this product presents lower risks to health than cigarettes”, and one substituting the word “lower” with “substantially lower” [17]. These two were compared to an HWL stating that snus is “not a safe alternative to smoking”. Smokers who saw the two comparative HWLs perceived daily use of snus as less harmful and reported that they were more likely to use and buy snus. For people who had tried or never used snus, seeing “substantially lower risk” was associated with lower risk perception of snus and the reporting of higher likelihood of buying snus. Another study assessed the effects of comparative risk information on actual snus use, although as a one-time provision of more extensive information instead of exposure to a brief HWL [18]. In this randomized trial, nicotine lozenge, snus, or snus combined with comparative risk information were tested as a means for smoking cessation, and cessation rates were found to be similar for all groups, with under 1.5% of the group participants staying abstinent for the whole year. The amount of snus used during cessation did not differ between the snus-only and the snus + comparative risk information group.

The studies above indicate that comparative HWLs can decrease the perception of risk from STLs relative to cigarettes. However, the studies represent only a limited number of contexts and only a few types of comparative health warnings, all of which are very general statements. The present study aimed to conceptually replicate the finding that general comparative health warnings can affect risk perceptions in a country with high prevalence of snus use, and to extend the research by investigating the impact of specific information about the relative risk. Specific versus general information have been found to be a relevant dimension in persuasion an advertisement research [19, 20].

In the present case, a general statement about less risk could mean slightly less risk, whereas a quantitative statement can better convey the magnitude of the difference in risk. In addition to the above, we were interested in whether a general non-comparative HWL, such as the one implemented in the EU, can distort the relative perceptions of risk from snus and smoking. That is, in comparison to a general statement regarding risk on snus packages, no information regarding risk may give perceptions that better reflect the large differences in risks between snus and smoking. A more detailed specification of our hypotheses follows.

### Hypotheses 1a and b (H1a and H1b)

The current non-comparative EU HWL states that snus “damages your health and is addictive,” a statement that is likely to produce an overestimation of the risks from snus use. We hypothesized that seeing a snus product with an HWL based on the current EU HWL would increase risk perception of snus use compared to seeing a control condition with no HWL (H1a), and that the relative perception of risk from snus use versus smoking would become lower in favor of snus use after exposure to the EU HWL compared to the control condition (H1b). Note that we chose to remove the “[…] *and is addictive*” statement from the EU HWL to make it similar to the below comparative risk HWLs.

### Hypotheses 2a and b (H2a and H2b)

Our first comparative HWL is a general claim that “Snus is less damaging to your health than smoking”, which we expected to adjust perceptions to reflect the differential health risks of snus and cigarettes more than would exposure to the EU-based HWL. Specifically, when compared to the EU-based HWL, this general comparative risk HWL (General CR) was expected to decrease perception of risk from snus (H2a), and to increase the difference between perceptions of risk from snus and smoking, such that the relative ratings are more in favor of snus use (H2b). Note that an increase in the (absolute) difference between snus use and smoking is reflected in a decrease in our relative score (snus risk minus smoking risk), lower scores indicating relatively less risk for snus use compared with smoking.

### Hypotheses 3a and b (H3a and H3b)

The second comparative HWL specifies that “Snus is 90% less damaging to your health than smoking.” This was based on a conservative estimate reported in a study by Levy et al. [21]. As hypothesized for the General CR condition, we expected risk perception of snus to decrease more in this percentage comparative risk (percentage CR) HWL condition than for the EU-based HWL (H3a), and that the difference in comparative risk would change more in favor of snus use in the percentage CR condition than in the EU-based HWL condition (H3b).

### Hypotheses 4a and b (H4a and H4b)

As the public tend to have unrealistically high estimates of risks from snus (3), the 90% difference in the percentage CR would likely be larger than most people would expect, and may, therefore, have a greater impact on perceptions than the general CR. Thus, the Percentage CR was expected to decrease risk ratings of snus more than the general CR (H4a), and to produce a stronger change in the relative perception of risk (H4b). For the sake of completeness, we decided a priori to include the analyses of Hypothesis 4b, but this hypothesis was not preregistered.

As intentions tend to be hard to affect [22] and our main focus was risk perception, we explored the effect of the HWLs on intentions to use tobacco and did not include these measures in the hypotheses or in the power calculation.

## Methods

### Participants

We collected the data in March 2019 in a convenience sample recruited from Facebook groups for Norwegian universities and www.slutta.no, a collection of official Norwegian resources about tobacco cessation. Of the 267 people who entered the survey, 254 completed it (95% completion rate, 69.3% females; age span 19–69, *M* = 36.39, SD = 11.92). Participants were required to be Norwegian speaking and over 16 years old. Based on the recruitment channel, we can assume that a majority of the participants have higher education, and that some of the participants have an interest in quitting using tobacco. People were encouraged to share the link to the survey. As the invitation was open, there is no guarantee that non-Norwegian speaking individuals have not participated. There was no payment for participation.

### Procedure

**Fig. 1:**
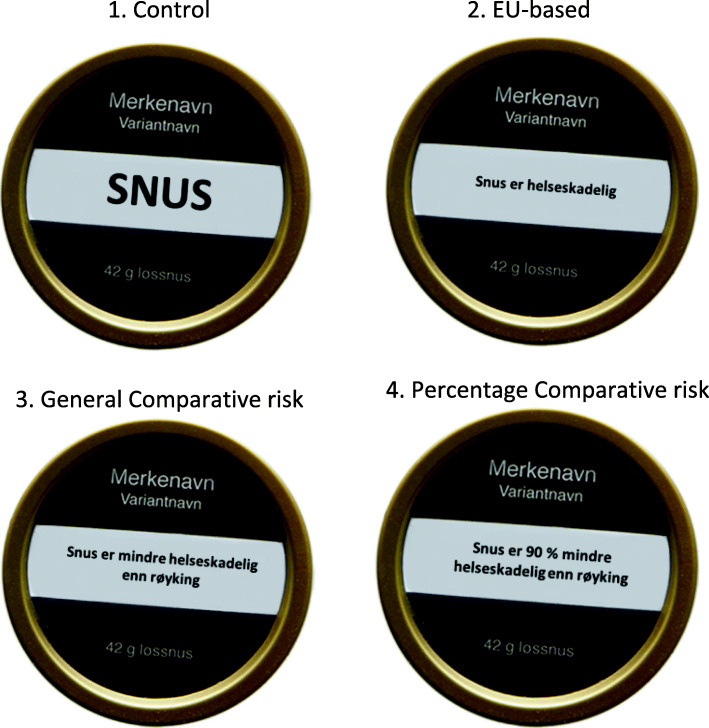
Health warning labels in the four conditions (original Norwegian versions)

Participants were instructed not to discuss details of the study on the Facebook invitation page and to avoid affecting how other people answered the questions. Participants were randomized to one of four conditions with different textual content (described above) on a snus package model: (1) control (no risk message), (2) EU-based HWL, (3) general comparative risk HWL (general CR), and (4) percentage comparative risk HWL (percentage CR). The snus package model was similar to the standardized packages used in Norway. The four HWLs (in the original Norwegian format) are presented in Fig. 1.

Risk perception and use intentions for both snus use and smoking were measured before (pre) and during (post) exposure to an HWL. Participants could view the picture of the snus package as long as they preferred while making the post-exposure ratings. It was not possible to go back to previous pages in the survey. The finishing page of the survey explained that the comparative risk HWLs were constructed for the study purpose and that the EU HWL was the approved version. Official resources with information about health risks from snus and smoking were made available. Demographics and current tobacco use were measured before the pre-exposure risk ratings. The hypotheses were preregistered at the Open Science Framework (https://osf.io/w8kp7/?view_only=9e7d405beb984df4b46bcb01e4e9602), and the data is available alongside this article (see Supplementary Material).

### Measures

Descriptive variables were age, gender, education, snus use, and smoking status. The measures of snus use and smoking status were categorized into non-users (never tried, quit, tried but no regular use) and users (sometimes or regularly).

We measured baseline risk perception of snus use and smoking in two separate questions: “Based on your current knowledge, how health damaging do you believe daily use of snus/smoking cigarettes is?” (Norwegian text: “Basert på den kunnskapen du har i dag, hvor helseskadelig tror du daglig snusbruk/daglig røyking av sigaretter er? ”). Participants responded on a Likert scale ranging from 1, not health damaging at all – 10, extremely health damaging. Although the scale has a semantic meaning, the numeric values are arbitrary. We nevertheless assumed that the scale approximated perceptions of the differences in risk (such that twice the rating would reflect a perception of twice the risk). We also included two questions about intention to use tobacco for exploratory analyses: “How likely are you to use snus/smoke cigarettes in the next six months?” (Norwegian text: “Hvor sannsynlig er det at du bruker snus/røyker sigaretter i løpet av de kommende 6 månedene?”), answered on a Likert scale ranging from 1, not likely at all – 10, extremely likely.

In the ratings following exposure to an HWL, we measured risk perception of snus use and smoking again: “When you see the snus package above, what are your thoughts about the health risks from daily use of snus?” (Norwegian text: “Når du ser snusboksen over, hva tenker du om helserisikoen ved å bruke snus daglig?”), and “What are your thoughts about the health risks from daily smoking of cigarettes?” (Norwegian text: “Hva tenker du nå om helserisikoen ved daglig røyking av sigaretter?”). Participants responded on a Likert scale ranging from 1, not health damaging at all – 10, extremely health damaging. Notice that the phrasing of the pre and post risk-measures of snus risks are slightly different. The pre-measure asks about current knowledge, and the post-measure about thoughts about risk after seeing the HWL. We are not interested in the changes per se, nor in the absolute levels of the ratings, but rather in the difference between the change in one condition and a change in a comparison condition which represent a counterfactual outcome (i.e., outcome if the participants in the former condition had received the same treatment as in the latter). This gives estimates of the causal effect of different treatments (HWLs) in comparison to each other. The post-exposure questions about intentions to smoke or use snus were identical to the baseline questions about intentions to use snus or smoke.

### Sample size

We conducted a within-subject pilot study where 40 respondents rated outcome measures from 6 HWLs (including the 3 in this study). The outcome measures in the pilot were designed to capture what the participants thought the HWLs were meant to convey. The differences in risk perception between two of the comparative risk HWLs were about Cohen’s *d* = 0.6. Because positive findings in pilot studies tend to give too high effect sizes [23], and because we made alterations in the outcome measures, we expected effect sizes of about 0.3. When including baseline ratings in the experimental design and assuming a correlation between pre and post measurement of *r* = 0.5, we would need around 50 persons in each condition for a power of 0.8 (*p* value threshold of .05). Thus, a total of 200 persons were needed in the present study. The data collection was closed when all groups had attained at least 50 participants.

### Statistical analysis

Each of the hypotheses were tested in separate repeated-measures analysis of variance (ANOVA) with HWLs as a between-group factor, pre- versus post-exposure as a within-group factor, and current snus use and smoking habits as covariates_._ Only the main effects of the tobacco use covariates were specified in the preregistration, but the interactions between covariates and within-subject factors are always included in the repeated general linear model function in IBM SPSS. In accordance with the pre-registered hypotheses, we only report inferential statistics for the interaction between the HWL conditions and the pre- to post-exposure measurements. This interaction represents the difference between the conditions in the change from pre- to post-exposure. For the hypotheses concerning comparative risk perception of snus use versus smoking, we constructed a new variable by subtracting the smoking rating from the snus rating. Note that the analyses could have been based on a single large model, with planned contrasts, but we found the present pairwise approach more transparent, and the separate models are also less restrictive than a full model. All tests were conducted with IBM SPSS 25.

## Results

Descriptive statistics of demographic variables according to the experimental condition are presented in Table 1. Means and standard deviations for risk perception rating pre- and post-HWL exposure are presented in Table 2.

**Table 1:** Demographics for each experimental condition

Demographic variable	Control (*n* = 50)	EU-based (*n* = 69)	General CR (*n* = 73)	Percentage CR (*n* = 75)
Age mean (SD)	33.50 (11.45)	37.18 (11.01)	36.61 (12.08)	37.41 (12.69)
Gender (%)				
Female	36 (72)	44 (63.8)	47 (64.4)	58 (77.3)
Male	14 (28)	23 (33.3)	26 (35.6)	17 (22.7)
Missing	0 (0)	2 (2.9)	0 (0)	0 (0)
Education (%)				
High school or less	23 (46)	19 (27.5)	28 (38.3)	32 (42.7)
Higher education	27 (54)	49 (71)	45 (61.6)	43 (57.3)
Missing	0 (0)	1 (1.4)	0 (0)	0 (0)
Snus habits (%)				
Never, tried, or quit	24 (48)	34 (49.3)	42 (57.5)	34 (45.3)
Sometimes or regularly	25 (50)	34 (49.3)	31 (42.5)	41 (54.7)
Missing	1 (2)	1 (1.4)	0 (0)	0 (0)
Smoking habits (%)				
Never, tried, or quit	40 (80)	61 (88.4)	66 (90.4)	64 (85.3)
Sometimes or regularly	9 (18)	8 (11.6)	6 (8.2)	11 (14.7)
Missing	1 (2)	0 (0)	1 (1.4)	0 (0)

*Note*. *SD* standard deviation

**Table 2:** Mean (standard deviations) for risk perception before and after exposure to warning labels

	Snus risk perception		Smoking risk perception		Perceived risk difference*	
	Pre	Post	Pre	Post	Pre	Post
Control	6.38 (2.20)	3.65 (2.79)	9.52 (0.81)	8.44 (2.44)	− 3.14 (2.06)	− 4.79 (3.34)
EU	5.58 (2.18)	5.33 (2.40)	9.26 (1.05)	8.88 (1.92)	− 3.66 (2.24)	− 3.55 (2.34)
Gen. CR	6.22 (2.48)	5.32 (2.38)	9.29 (1.47)	9.22 (1.64)	− 3.07 (2.42)	− 3.90 (2.46)
% CR	5.87 (2.42)	3.86 (2.02)	9.20 (1.25)	9.41 (1.17)	− 3.32 (2.26)	− 5.54 (2.20)

*Note*. Responses were made on 10-point risk scales from 1, “not at all” to 10 “extremely”.

*Perceived risk difference was calculated by subtracting the smoking rating from the snus rating

### Differences in changes in risk perception after HWL exposure

In terms of the direction of the effects, all the preregistered hypotheses (H1a, H2a, H3a, H4a) about between-condition differences in the changes in perception of health risk from snus were supported, but the statistical evidence for H2a was weak. All of the hypotheses about changes in the perceived risk difference (H1b, H2b, H3b, and H4b) were supported.

In the comparison of the EU-based HWL and the control condition, there was only a slight change for the EU HWL (*M*_Pre_ = 5.64 vs. *M*_Post_ = 5.33) versus a marked decrease for the control condition (*M*_Pre_ = 6.33 vs. *M*_Post_ = 3.65). This interaction was statistically significant, *F*(1, 110) = 25.04, *p* < .001, η_p_^2^ = .19. Thus, in relative terms, the EU HWL showed an increase in risk perception over the control condition (considering the control condition as the counterfactual outcome). This result confirmed H1a although in a slightly different way than we expected. We believed the EU HWL would increase and the control to remain stable, but note that the individual changes from the pretest are not important for the inference of a difference between the conditions. Similarly, H1b was confirmed, as exposure to the EU-based HWL maintained perceived risk difference levels from pre to post (*M*_Pre_ = − 3.62 vs. *M*_Post_ = − 3.55), while the control condition decreased the risk difference ratings of snus use (*M*_Pre_ = − 3.17 vs. *M*_Post_ = − 4.79), *F*(1, 110) = 15.49, *p* < .001, η_p_^2^ = .12.

In the next hypothesis (H2a), we expected the General CR (*M*_Pre_ = 6.25 vs. *M*_Post_ = 5.32) to decrease perception of risk more than the EU HWL (*M*_Pre_ = 5.64 vs. *M*_Post_ = 5.33). The perception estimates decreased slightly in descriptive terms, although there was no statistically significant interaction effect, *F*(1, 133) = 2.42, *p* = .122, η_p_^2^ = .02. Seeing the general CR (*M*_Pre_ = − 3.13 vs. *M*_Post_ = − 3.97) produced a slightly stronger decrease in the measure of perceived risk differences in comparison to seeing the EU HWL (H2b) (*M*_Pre_ = − 3.62 vs. *M*_post_ = − 3.55), *F*(1, 133) = 7.64, *p* = .007, η_p_^2^ = .05.

The expectation that the percentage CR would decrease the perception of risk more than the EU-based HWL (H3a) was supported, as the EU version decreased less than half a point (*M*_Pre_ = 5.64 vs. *M*_Post_ = 5.33) whereas the percentage CR decreased more than two points (*M*_Pre_ = 5.92 vs. *M*_Post_ = 3.86). This interaction effect was statistically significant, *F*(1, 136) = 23.24, *p* < .001, η_p_^2^ = .15. Thus, concrete relative risk information in the HWL lowered risk estimates from snus more than the EU-based version did.

Also the perceived risk difference scores decreased more in the percentage CR (*M*_Pre_ = − 3.32 vs. *M*_Post_ = − 5.52) than in the EU-based HWL (*M*_Pre_ = 3.62 vs. *M*_Post_ = − 3.55), *F*(1, 135) = 47.85, *p* < .001, η_p_^2^ = .26. This supported our hypothesis H3b. The differences in change were the largest in this comparison, with the percentage CR HWL producing a marked drop in risk difference ratings compared to the EU-based HWL.

The percentage CR with concrete information about the relative risk was expected to decrease risk ratings from snus use more than the General CR (H4a). This hypothesis was supported, *F*(1, 141) = 13.21, *p* < .001, η_p_^2^ = .09. The General CR decreased ratings with almost one point (*M*_Pre_ = 6.25 vs. *M*_Post_ = 5.32) whereas the percentage CR decreased over two points (*M*_Pre_ = 5.92 vs. *M*_Post_ = 3.86). Similarly, the percentage CR (*M*_Pre_ = − 3.32 vs. *M*_Post_ = − 5.52) decreased the perceived risk difference scores more than the general CR did (*M*_Pre_ = − 3.13 vs. *M*_Post_ = − 3.97), *F*(1, 140) = 15.90, *p* < .001, η_p_^2^ = 10.

### Intentions to use snus

Effects of seeing an HWL on the intention to use snus were explored for all HWLs in one model. We did not test intentions to smoke because there were too few smokers in the sample. There was no interaction effect between pre- and post-measurement and the HWLs in terms of intentions to use snus, *F*(3, 252) = .40, *p* = .75, η_p_^2^ = .01, but already using snus was associated with having stronger future intentions to use snus (around 8.5 on the scale from 1 to 10) than not using snus (around 1.5 on the scale), *F*(1, 252) = 698.57, *p* = .000, η_p_^2^ = .76.

## Discussion

Norwegian participants recruited through social media were randomized to see one of four HWLs and rated risk perceptions of tobacco use pre- and post-HWL exposure. All the preregistered hypotheses were supported. In comparison to the non-comparative EU-based warning, the other comparative risk HWLs, as well as no HWL, lowered the perception of risk from snus and changed the risk ratings in favor of snus use (lowering the perceived risk from snus use relative to smoking). These results conform to expectations, as we hypothesized that the EU-based HWL would produce a higher perception of risk from snus than the CR HWLs because it is an absolute statement focusing on the harm of snus, with no reference to more harmful tobacco products that could have provided perspective.

The statistical evidence for the predicted difference between the EU-based HWL and the general CR HWL was weak. Although there was a slight tendency in the expected direction as the EU HWL maintained high risk estimates from snus while the General CR HWL decreased it, the changes were fairly similar for these two HWLs. The general CR HWL is a quite imprecise claim that snus is “less damaging to your health” than smoking. “Less” is an abstract term with a broad range, and may not activate the idea that the risk from snus can be substantially lower than originally assessed. However, perception of risk from snus relative to smoking differed for the EU-based and General CR HWLs, with the latter HWL increasing the relative estimate differences more, as expected. This conceptually replicates the effect in the study on young Canadians, where the general comparative HWL “Using ST is less harmful than smoking cigarettes” was found to lower relative risk estimates [16].

As expected, a concrete percentage format for the relative risk had a stronger effect than the general statement on lowering the perception of risk from snus, and in increasing the difference between snus use and smoking such that the relative perceptions of risk were more in favor of snus. A possible explanation for our result is that with risks from snus generally being exaggerated when compared to cigarettes [3], reading a statement that snus is 90% less health damaging can have a strong impact. Although the study by Rodu et al. [17] used non-specific comparative HWLs, their modifier “substantially” (i.e., “[…] substantially lower risks[…]”, likely produced a similar effect by emphasizing the magnitude of risk differences.

Popova and Ling [24] found that more people rated snus use as less harmful than cigarettes when the risks were measured and compared indirectly from two separate questions rather than directly from one single question (51.6% versus 22.1%). Thus, the indirect comparison of snus and smoking we required in two separate questions may have reduced some of the overrating of risks from snus that could have been generated from one single item of relative risk and potentially produced more realistic risk ratings.

One may have expected no change from pre to post in the control group, as this group was not exposed to any HWL but to a snus product with the text “Snus” only. Although it is difficult to interpret changes per se due to the different types of questions pre and post exposure (general knowledge of risk versus perception of risk from exposure), it is interesting to note that the perception of risk from snus decreased the most in the control group. One may speculate that exposure to products with no HWL gives a more realistic perception of risk, and that any textual HWLs, whether comparative or non-comparative, increase risk perception in comparison to no information. However, this reasoning is based on post hoc observations of the data and the study was not designed to test these ideas.

Intentions to use snus did not vary between HWLs, but it is likely that we did not have sufficient power to test effects on intentions. If intentions remained stable, this could be a desirable quality of the HWLs. The sample consisted mainly of non-smokers, and their interest in using snus did not increase, even though their risk perceptions changed in favor of snus. Other literature indicates that there is an association between heightened risk perception and behavior [22], and some studies have found effects from comparative risk information on behavioral variables, e.g., [16, 17], while other studies have not [18].

## Limitations

There are limitations to our study. First, females are overrepresented in the sample, possibly limiting the generalizability of findings. Furthermore, there were slight differences in the phrasings of pre and post risk measures, which means that we can only interpret the differences in changes, not the absolute levels, or the changes within conditions. The differences we found between our conditions were produced by a one-time exposure to an online picture of a snus product with an HWL and may not be generalizable to real-life exposure or have any long-term impact. Participants were asked to rate their perceived risk while looking at the HWL, but this perception may change when the HWL is no longer present. Furthermore, the short time between pre and post measures may have primed the perceptions and affected the results. We used self-reported measures, which can be biased if participants moderate their answers to be more socially favorable, as described by the social desirability bias [25]. The scale could have been clearer defined, such that we could know more exactly how people assessed risk (e.g., in terms of number of diseases per 1000 or mortality rate). Finally, as our recruitment was done via an open invitation link on Facebook, we do not have much information about the gross sample, for example of who saw the invitation but chose not to respond. This implies that we do not know how representative our sample is for the general population.

## Conclusion

Comparative risk information in snus HWLs reduces perceived risk from snus use and adjusts relative perceptions of risk between snus and smoking. At least in the case of products that differ greatly in risk, a message that frames the relative risk in terms of percentages can be considerably more potent than a general comparative risk claim.

## Data Availability

The dataset supporting the conclusions of this article will be made available in the UiT Open Research Data repository, along with publication of the manuscript.

## Abbreviations

ANOVA: Analysis of variance
EU: European Union
HWL: Health Warning Label
WHO: World Health Organization

## References

[1] The U.S. Food and Drug Administration. FDA News release: FDA grants first-ever modified risk orders to eight smokeless tobacco products. 2019. https://www.fda.gov/news-events/press-announcements/fda-grants-first-ever-modified- risk-orders-eight-smokeless-tobacco-products. .

[2] Wackowski OA, Ray AE, Stapleton JL (2019). Smokers' perceptions of risks and harm from snus relative to cigarettes: A latent profile analysis study. Add Behav..

[3] Lund EK, Vedoy FT. Relative risk perceptions between snus and cigarettes in a snus- prevalent society—an observational study over a 16 year period. Int J Environ Res Public Health. 2019;16(5). 10.3390/ijerph16050879 PMC6427464 30862006

[4] Lund I, Scheffels J (2014). Perceptions of relative risk of disease and addiction from cigarettes and snus. Psychol Addict Behav..

[5] WHO Framework Convention on Tobacco Control. Guidelines for implementation of Article 11 of the WHO Framework Convention on Tobacco Control on “Packaging and labelling of Tobacco Products” (decision FCTC/COP3(10)) 2003. 2003. http://www.who.int/fctc/text_download/en/. Accessed 17 June 2019.

[6] Near AM, Blackman K, Currie LM, Levy DT (2014). Sweden SimSmoke: the effect of tobacco control policies on smoking and snus prevalence and attributable deaths. Eur J Public Health..

[7] Hammond D (2011). Health warning messages on tobacco products: a review. Tob Control..

[8] EUR-LEX. Council directive 92/41/EEC of 15 may 1992 amending directive 89/622/EEC on the approximation of the laws, Regulations and administrative provisions of the member states concerning the labelling of tobacco products. 1992. http://eur-lex.europa.eu/legal-content/en/ALL/?uri=CELEX%3A31992L0041. Accessed 12 Feb 2020.

[9] EUR-LEX. Directive 2001/37/EC Manufacture, Presentation and sale of tobacco products. 2001. https://eur-lex.europa.eu/legal-content/EN/TXT/?uri=LEGISSUM%3Ac11567. Accessed 12 Feb 2020.

[10] EUR-LEX. Directive 2014/40/EU of the European Parliament and of the Council of 3 April 2014 on the approximation of the laws, regulations and administrative provisions of the Member States concerning the manufacture, presentation and sale of tobacco and related products and repealing Directive 2001/37/EC Text with EEA relevance 2014. 2014. http://eur-lex.europa.eu/legal-content/EN/TXT/?uri=celex:32014L0040. Accessed 17 June 2019.

[11] Nilsen CV, Friborg O, Teigen KH, Svartdal F (2018). Textual health warning labels on snus (Swedish moist snuff): do they affect risk perception?. BMC Public Health..

[12] Kozlowski LT, Sweanor D (2016). Withholding differential risk information on legal consumer nicotine/tobacco products: The public health ethics of health information quarantines. International J Drug Policy..

[13] Kaufman AR, Suls JM, Klein WM (2016). Communicating tobacco product harm: Compared to what?. Addict Behav..

[14] Levy DT (2018). Communicating accurate and complete information. Addict Behav..

[15] Levy DT, Mays D, Boyle RG, Tam J, Chaloupka FJ. The effect of tobacco control policies on US smokeless tobacco use: a structured review. Nicotine Tob Res. 2017;13:20(1):3-11. 10.1093/ntr/ntw291 PMC5896466 27798090

[16] Callery WE, Hammond D, O'Connor RJ, Fong GT (2011). The appeal of smokeless tobacco products among young Canadian smokers: The impact of pictorial health warnings and relative risk messages. Nicotine Tob Res..

[17] Rodu B, Plurphanswat N, Hughes JR, Fagerstrom K (2016). Associations of proposed relative- risk warning labels for snus with perceptions and behavioral intentions among tobacco users and nonusers. Nicotine Tob Res..

[18] Nelson PR, Chen P, Battista DR, Pillitteri JL, Shiffman S (2019). Randomized trial to compare smoking cessation rates of snus, with and without smokeless tobacco health-related information, and a nicotine lozenge. Nicotine Tob Res..

[19] O'Keefe DJ (1997). Standpoint explicitness and persuasive effect: A meta-analytic review of the effects of varying conclusion articulation in persuasive messages. Argumentation and Advocacy..

[20] Feldman DC, Bearden WO, Hardesty DM (2006). Varying the content of job advertisements: The effects of message specificity. J Advert..

[21] Levy DT, Mumford EA, Cummings KM, Gilpin EA, Giovino G, Hyland A (2004). The relative risks of a low-nitrosamine smokeless tobacco product compared with smoking cigarettes: estimates of a panel of experts. Cancer Epidemiol Biomarkers Prev..

[22] Sheeran P, Harris PR, Epton T (2014). Does heightening risk appraisals change people’s intentions and behavior? A meta-analysis of experimental studies. Psychol Bull..

[23] Albers C, Lakens D (2018). When power analyses based on pilot data are biased: Inaccurate effect size estimators and follow-up bias. J Exp Soc Psychol..

[24] Popova L, Ling PM (2013). Perceptions of relative risk of snus and cigarettes among US smokers. Am J Public Health..

[25] Fisher RJ (1993). Social desirability bias and the validity of indirect questioning. J Consum Res..

